# The effect of pH on the chemical and structural interactions between apple polyphenol and starch derived from rice and maize

**DOI:** 10.1002/fsn3.1800

**Published:** 2020-07-29

**Authors:** Shurui Chou, Bin Li, Hui Tan, Weijia Zhang, Zhihuan Zang, Huijun Cui, Hanchen Wang, Shuyi Zhang, Xianjun Meng

**Affiliations:** ^1^ College of Food Science Shenyang Agricultural University Shenyang China

**Keywords:** apple polyphenol, pH conditions, starch

## Abstract

To date, how pH affects starch–polyphenol mixtures has not been thoroughly investigated. This study explored the impact of combining apple polyphenol (AP) with both normal rice starch (NRS) and normal maize starch (NMS) across a range of pH conditions. NRS–AP mixture particle sizes across a pH range of 3–8 varied from 169.9 ± 5.4 to 187.5 ± 6.9 μm, while for NMS–AP particles, these sizes ranged from 161.8 ± 8.0 to 176.0 ± 4.9 μm, indicating that the aggregation of starch–AP was inhibited under low pH condition. The melting enthalpy (△H) values of the NRS–AP mixture across a pH range of 3–8 were 8.50 ± 0.06–9.56 ± 0.12 J/g, while the corresponding value for the NMS–AP mixture was 5.77 ± 0.05–6.21 ± 0.08 J/g. FTIR analyses revealed that the degree of order of these starch–AP mixtures significantly decreased under low pH conditions. XRD analysis further revealed that both NRS–AP and NMS–AP mixtures exhibited V‐type structures, and relative crystallinity levels decreased significantly under low pH conditions. Together, these results indicate that low pH values inhibit the recrystallization of NRS–AP and NMS–AP mixtures. Overall, these findings provide additional evidence regarding the interactions between AP and specific starches under a range of pH conditions.

## Graphical abstract

Rice/maize starch–apple polyphenol (RS–AP and MS–AP) mixtures were prepared. The structures of RS–AP and MS–AP mixtures were observed.

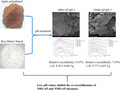

**Highlights**

- Rice/maize starch‐apple polyphenol (NRS–AP and NMS–AP) mixtures were prepared.
- Low pH values inhibit the recrystallization of NRS–AP and NMS–AP mixtures.
- Low pH values decrease the order of NRS–AP and NMS–AP mixtures.

## 1 INTRODUCTION

Starches are naturally formed polysaccharide macromolecules that are among the most common forms of energy storage in nature, serving as abundant and biodegradable compounds that have no associated risk of direct environmental pollution (Tester, Karkalas, & Qi, 2004). As they are the primary mechanism whereby plants store solar energy that has been converted into chemical energy, starches are a main energy source for most animal species including humans, and represent an effectively inexhaustible natural resource (Burrell, 2003; Zhang, Zhang, Xu, Li, & Tan, 2018; Zhang, Zhu, He, Tan, & Kong, 2015). Importantly, starch molecules are highly amenable to chemical modification as the reactive OH groups within the glucose portions of these starch molecules can be exchanged for alternate chemical groups and molecules (Singh, Kaur, & McCarthy, 2007).

In the food production industry, starches are frequently used as an alternative to fats in the production beverages, salad dressings, and ice cream owing to the fact that upon mixing with they form a gel‐like mixture with creamy textural properties (Amagliani, O'Regan, Kelly, & O'Mahony, 2016). However, the intrinsic utility of starches for the production of chemicals, medicines, or food products is limited by the fact that these starches tend to exhibit poor solubility in cold water. As the combination of starches with specific substances can overcome these limitations (Thomas & Atwell, 1999 Sikora & Kowalski, 2003), many researchers have explored the molecular interactions between starches and other macromolecules including lipids, proteins, and other polysaccharides. When mixed with proteins, starch and protein molecules have been shown to interact through both covalent and noncovalent bonding, as well as through molecular entanglement and volume exclusion (Fernández‐Gutiérrez, Martín‐Martínez, Martínez‐Bustos, & Cruz‐Orea, 2010; Guan, Qiu, Liu, Hua, & Ma, 2006; Kato, Mifuru, Matsudomi, & Kobayashi, 2008; Zaleska, Ring, & Tomasik, 2001). In contrast, interactions between starches and lipids occur primarily as a result of hydrophobic interactions between lipid chains and the nonpolar regions of starch molecules, resulting in the lipid molecules localizing within the hydrophobic spiral structures of these starch molecules (Chang, He, & Huang, 2013). Interactions between starches and other polysaccharides have been shown to mediate a significant increase in the viscosity of a given system, thereby significantly impairing the time‐dependent breakdown of the resultant solution (BeMiller, 2011).

Polyphenols are highly prevalent in nature which offer a number of beneficial health effects, and can be used to directly facilitate food or beverage production. As such, there has been increasing interest in recent years regarding interactions between starches and polyphenols. The application of such starch–polyphenol complexes is promising, and many researchers have intensively researched approaches to reducing starch digestion. Liu, Wang, Peng, and Zhang (2011) found that a combination of tea polyphenols and maize starch was sufficient to regulate postprandial blood glycemic responses. Barros, Awika, and Rooney (2012) used sorghum extracts to combine with maize starch in order to increase the resistant starch in maize starch, while kaempferol–starch complexes have been prepared to decrease starch digestion and order of amylose (Takahama & Hirota, 2013).

Several recent studies have explored the impact of polyphenols on the physicochemical and structural properties of starchy foods (Barros et al., 2012; Guzar, Ragaee, & Seetharaman, 2012; Hung, Phat, & Phi, 2013; Zhu, 2015). Noncovalent interactions exist between starches and polyphenols, and XRD analyses have revealed that V‐type inclusion complexes may be formed in starch–polyphenol mixtures. The self‐assemble of these starch–polyphenol complexes can also be mediated via hydrogen bonding (Chai, Wang, & Zhang, 2013). Polyphenols can affect starch retrogradation and gelatinization, with black tea extracts having been shown to increase the gelatinization temperature of potato starch (Xiao, Lin, Liu, & Yu, 2012), while epigallocatechin gallate decreased the gelatinization temperature of potato, maize, and rice starches (Wu, Chen, Li, & Li, 2009), indicating that different types of polyphenols have diverse effects on starches.

How different pH levels impact the physicochemical and structural properties of starch–apple polyphenol mixtures has not been examined. In the present study, we utilized a mixture of apple polyphenol (AP) and either normal rice or maize starch (NRS and NMS, respectively), as these compounds are commonly used for food production. We prepared these NRS–AP and NMS–AP mixtures under different pH conditions in order to assess how pH affected the interaction between AP and starch molecules, as a thorough understanding of such interactions is essential to reliable food production.

## 2 MATERIALS AND METHODS

### 2.1 Materials

AP (95.0% pure) was obtained from Hubei Jusheng Technology Co., Ltd. (Wuhan, Hubei, China). This mixture was composed of ~43.3% procyanidins, ~20.0% chlorogenic acid, ~11.7% phlorizin and phloretin, ~5.4% anthocyanins, ~4.7% [−]‐epicatechin, [+]‐catechin and gallic acid, ~3.4% p‐coumaric acid, and ~3% hydrochalcone (≈3%). NMS (moisture content: 18.07%, apparent amylose content: 25.5%) and NRS (moisture content: 15.35%, apparent amylose content: 21.2%) were from Dingguo Biological Technology Co., Ltd. (Shenyang, Liaoning, China). Methyl silicone oil, KBr, HCl, and NaOH were from Sinopharm Chemical Reagent Co., Ltd.

### 2.2 Starch–AP mixture preparation

To prepare the samples of starch–AP mixtures, 50 mg AP was combined with 1 g of the indicated starch (NRS and NMS) in a 20 ml volume of different pH (3.0, 4.0, 5.0, 6.0, 7.0, and 8.0) solutions in a 95°C water bath with constant stirring for 20 min. After preparation was completed, starch–AP mixtures were slowly cooled to room temperature prior to lyophilization.

### 2.3 Measurement of size distributions

A laser scattering particle analysis instrument (BT‐9300S, Dandong Bettersize Instruments Ltd.) was used to assess particle sizes in the indicated starch–AP mixtures. Briefly, sample mixture scattering was conducted in deionized water, with 3 min of ultrasonication being used to ensure that the particles were dispersed evenly. Particle size was then measured at an obscuration level of 16.39%. The experiment was repeated thrice, with average values being determined. Starch granules and water had respective refractive index values of 1.60 and 1.333.

### 2.4 X‐ray diffraction (XRD)

An X‐ray diffraction device (Bruker AXS, Bruker Corporation) was used for XRD image generation at 40 kV and 40 mA. Starch–AP mixtures were subjected to a continuous scan with a 2θ limit from 5° to 40°. A 2°/min scanning speed was utilized, with a 0.02° step length. The relative crystallinity values of samples were calculated as the ratio of crystallized area to the total area by Jade 5.0.

### 2.5 Fourier‐transform infrared (FTIR) spectroscopy

Initially, 2 mg of the indicated starch–AP samples was mixed together with 198 mg KBr powder prior to compression. An FTIR spectrometer (Thermo Fisher Scientific) was then used to analyze samples at room temperature within a 4,000–400 cm^−1^ wavelength range, with 16 scans per spectrum being collected at a 4 cm^−1^ resolution ratio.

### 2.6 Differential scanning calorimetry (DSC) analysis

DSC results were obtained with an appropriate DSC instrument (Pyris‐1, Perkin‐Elmer Co.) by the method of Liu et al. (2017) described with minor modification. Briefly, a total of 3 mg of the indicated mixture was added to the steel pan of the instrument and was evenly dispersed in deionized water at a 1:2 weight ratio of sample to water. Samples were then held at 25°C for 1 min prior to being heated to 95°C at 10°C/min. Samples were then stored at 4°C for 7 days and were then tested again via heating from 25°C to 95°C.

### 2.7 Scanning electron microscopy (SEM)

Starch–AP mixture morphology was analyzed via SEM using an EVO MA 15 microscope (ZEISS). Briefly, the indicated sample mixtures underwent freeze‐drying, mounting, and vacuum sputtering‐mediated plating with a layer of gold. SEM was conducted using a 10 kV acceleration voltage and a magnification level of 500×.

### 2.8 Statistical analysis

All the measurements were repeated three times, and the data were analyzed with analysis of variance (ANOVA) and shown as mean ± *SD*. SPSS 18.0 (IBM) and MATLAB 2014Ra (MathWorks) were used to analyze all results. Duncan tests were conducted, and the significance difference was represented at *p* < .05.

## 3 RESULTS AND DISCUSSION

### 3.1 Particle distribution

We began by assessing NMS–AP and NRS–AP mixture particle sizes under a range of pH conditions (pH 3.0–8.0). We found that the addition of AP resulted in marked changes in NRS and NMS particle size, as shown in Table 1. Specifically, the NRS, NRS–AP, NMS, and NMS–AP samples had particle size values of 71.2 ± 3.6 μm, 197.7 ± 1.5 μm, 42.6 ± 2.9 μm, and 183.0 ± 6.2 μm, respectively. Phenolic acids of differing molecular weights and structures have previously been reported to increase the size of starches via mediating large aggregate formation (Concha et al., 2018). We found that both particle sizes of NRS–AP and NMS–AP mixture at low pH conditions were lower than those at high pH conditions. The NRS–AP and NMS–AP samples had particle size values of 169.9 ± 5.4 μm and 161.8 ± 8.0 μm, respectively. These results, therefore, indicated that low pH influences starch–AP mixture particle size significantly. Previous reports have found that the combination of (+)‐catechin (C) and proanthocyanidin (PAC) with modified waxy corn starch under alkaline conditions resulted in a larger particle size distribution than under acid conditions. In contrast, this study did not observe any effect of acidic or alkaline treatment on complexes of (‐)‐epicatechin (EC) and waxy corn starch, and the combination of (−)‐epigallocatechin‐3‐gallate (EGCG) with waxy corn starch exhibited a greater particle size distribution under alkaline conditions than under acidic ones (Liu et al., 2016). These variations were primarily attributable to the structures of these phenolic compounds and the resultant changes in their ability to bind to starch, with additional effects being due to the impact of pH on the structure of these polyphenols. The authors of this previous study also speculated that particle charge may impact the influence of pH on polyphenol–starch complexes (Liu et al., 2016).

**TABLE 1:** (a) Particle size results for NRS and NRS–AP mixtures under various pH treatment conditions. (b) Particle size results for NMS and NMS–AP mixtures under various pH treatment conditions

Sample	Particle size (μm)
(a)	
NRS	
Raw starch	71.2 ± 3.6a
NRS–AP	
Without pH treatment	197.7 ± 1.5d
pH 3	169.9 ± 5.4b
pH 4	178.3 ± 6.2bc
pH 5	180.9 ± 5.6bc
pH 6	183.9 ± 8.8c
pH 7	187.5 ± 6.9c
pH 8	184.7 ± 9.6c
(b)	
NMS	
Raw starch	42.6 ± 2.9a
NMS–AP	
Without pH treatment	183.0 ± 6.2b
pH 3	161.8 ± 8.0b
pH 4	162.5 ± 6.7b
pH 5	172.7 ± 5.5b
pH 6	171.2 ± 7.6c
pH 7	176.0 ± 4.9c
pH 8	170.4 ± 3.7c

Note

Values with the same letters in the same column do not differ significantly (*p* < .05).

### 3.2 DSC analysis

The thermal properties of NRS, NMS, NRS–AP, and NMS–AP were next analyzed (Table 2). AP did not have a significant impact on peak temperature (Tp) or melting enthalpy (△H) for NRS, whereas AP did significantly alter these values for NMS. Tp values for NRS, NRS–AP, NMS, and NMS–AP were 77.24 ± 0.05, 77.04 ± 0.33, 78.54 ± 0.05, and 81.09 ± 0.05℃, respectively, while the corresponding △H values were 9.79, 9.56, 7.19, and 6.28 J/g, respectively. Differences in results between studies likely arose as a consequence of the different molecular structures of these tested starches, leading them to undergo different molecular interactions with AP.

**TABLE 2:** (a) Thermal properties of NRS–AP mixture with different pH treatment. (b) Thermal properties of NMS–AP mixture with different pH treatment

Samples	To (°C)	Tp (°C)	Tc (°C)	△H (J/g)
(a)				
NRS				
Raw starch	72.97 ± 0.10d	77.24 ± 0.05b	84.68 ± 0.06e	9.79 ± 0.24d
NRS–AP				
Without pH treatment	72.57 ± 0.04c	77.04 ± 0.33b	81.08 ± 0.10a	9.56 ± 0.21cd
pH 3	73.40 ± 0.13e	76.64 ± 0.08a	84.57 ± 0.11e	8.50 ± 0.06a
pH 4	72.13 ± 0.11a	76.66 ± 0.03a	81.55 ± 0.06b	9.20 ± 0.15b
pH 5	72.34 ± 0.09b	77.02 ± 0.05b	81.81 ± 0.03c	9.49 ± 0.04c
pH 6	73.64 ± 0.09f	79.75 ± 0.02c	84.60 ± 0.05e	9.56 ± 0.12cd
pH 7	77.87 ± 0.08h	80.16 ± 0.11d	83.60 ± 0.11d	9.54 ± 0.07cd
pH 8	75.41 ± 0.06g	79.96 ± 0.03d	83.68 ± 0.23d	9.33 ± 0.05bc
(b)				
NMS				
Raw starch	74.01 ± 0.12b	78.54 ± 0.05a	88.34 ± 0.03g	7.19 ± 0.09e
NMS–AP				
Without pH treatment	77.05 ± 0.03f	81.09 ± 0.05d	87.78 ± 0.04e	6.28 ± 0.04d
pH 3	74.45 ± 0.07c	80.60 ± 0.08b	88.57 ± 0.04h	5.77 ± 0.05a
pH 4	77.81 ± 0.18g	80.83 ± 0.02cd	87.89 ± 0.07f	6.02 ± 0.24b
pH 5	75.80 ± 0.12e	81.12 ± 0.22d	87.34 ± 0.03d	6.14 ± 0.22bc
pH 6	79.40 ± 0.25h	80.94 ± 0.10c	86.62 ± 0.07b	6.21 ± 0.08bc
pH 7	75.10 ± 0.06d	81.82 ± 0.27e	87.14 ± 0.08c	6.27 ± 0.04bc
pH 8	72.83 ± 0.14a	80.72 ± 0.05bc	83.50 ± 0.06a	6.16 ± 0.12bc

Note

Values with the same letters in the same column do not differ significantly (*p* < .05).

The △H values of both the NRS–AP and NMS–AP mixtures at low pH conditions were lower than those at high pH conditions (Table 2). △H reflects the loss of the double‐helical order of starch (Cooke & Gidley, 1992). As such, this result indicates that low pH values inhibited the retrogradation of NRS–AP and NMS–AP mixtures. This may be a result of the fact that acidic conditions depolymerize starch–AP molecules, thereby reducing the amount of energy needed for starch molecules to reach Tp. The △H values of both the NRS–AP and NMS–AP mixtures at pH 8 were lower than those at pH 6 and pH 7. It is maybe that the presence of additional hydroxyl ions can reduce interactions between water molecules under alkaline condition, thereby making it easier for these molecules to enter into the interior of starch molecules and thus allowing for a more effective combination of starch and AP molecules, leading to reducing the amount of energy needed for starch molecules to reach Tp. As additional water molecules enter into these starch molecules, complete gelatinization occurs and the energy required for this reaction rises, thereby increasing △H (Xie, Xiao‐Fang, Xiao, & Liu, 2009).

### 3.3 XRD analysis

**FIGURE 1:**
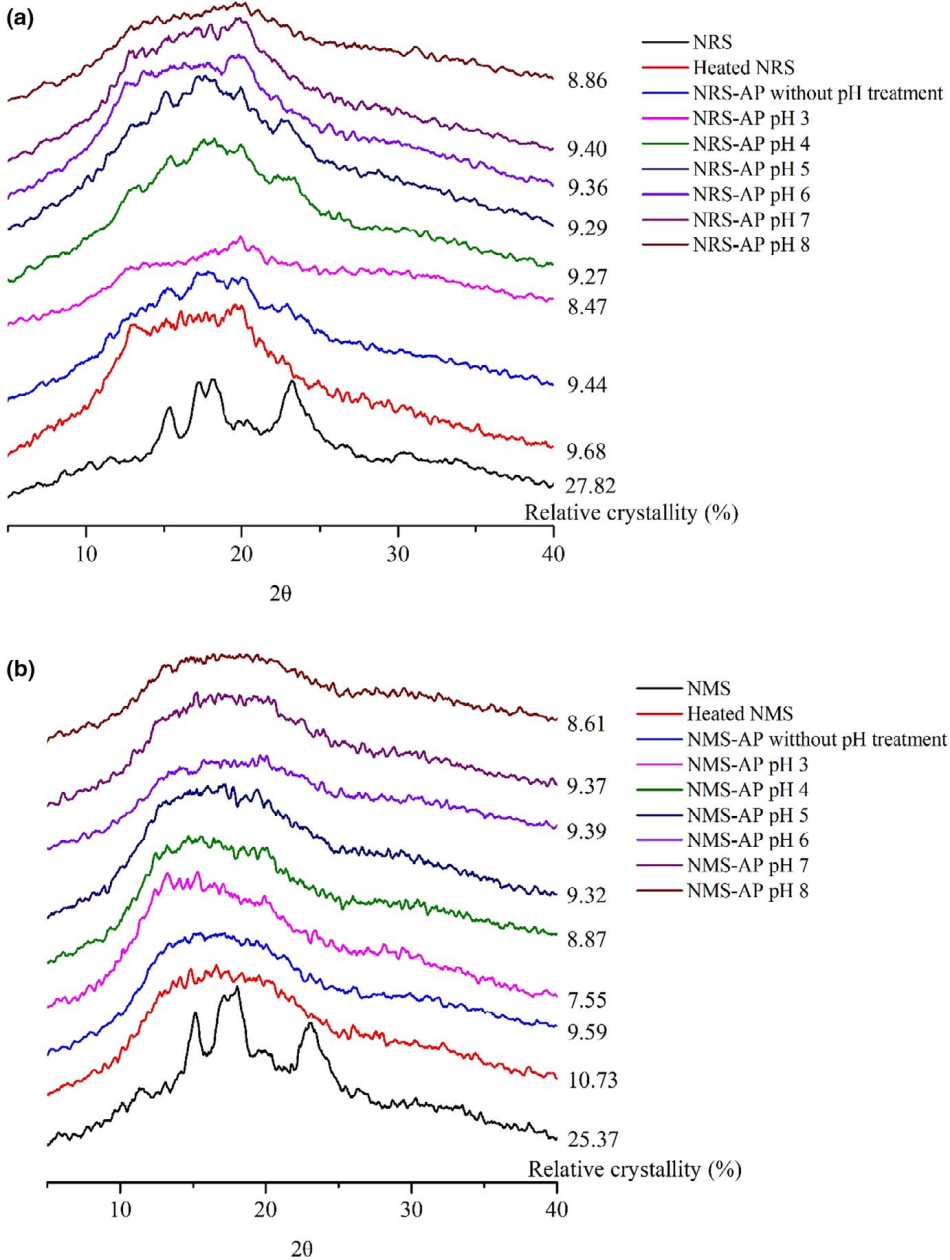
(a) X‐ray diffraction results for NRS–AP mixtures under the indicated pH treatment conditions; (b) X‐ray diffraction results for NMS–AP mixtures under the indicated pH treatment conditions

XRD was used to assess changes in the crystallinity of starch–AP mixtures as a function of pH (Figure 1). Diffraction patterns revealed high diffraction peaks for both NRS and NMS at 2θ = 15°, 18°, and 23°, indicative of a typical A‐type structure (Banks & Greenwood, 1975; Jeong & Shin, 2018). In contrast, the starch–AP mixtures exhibited strong diffraction peaks at 12.8°, 13°, and 20° of 2θ, consistent with a typical V‐type structure resulting from the entry of AP into the spiral structure of the starch molecule.

Due to heating, the starch molecules underwent a transition from a crystalline to an amorphous state, and the relative crystallinity of the starch decreased. When AP was added to starch, AP competed with water molecules and interacted with starch chains, resulting in decreased particle swelling and the repolymerization of starch chains, leading to an decrease in the relative crystallinity of the starch–AP mixture. The effect of pH values on these starch –polyphenol complexes was thus significant. At acidic condition, the crystalline structure of starch was disrupted (Lee, Lee, & Lee, 2018) and recrystallization was also inhibited, whereas relative crystallinity rose as pH values increased because the crystalline structure was not disrupt obviously under higher pH conditions (pH < 7). At alkaline condition, starch granules were swelled and broken, leading to an decrease in the relative crystallinity of the starch–AP mixture.

### 3.4 FTIR analysis

**FIGURE 2:**
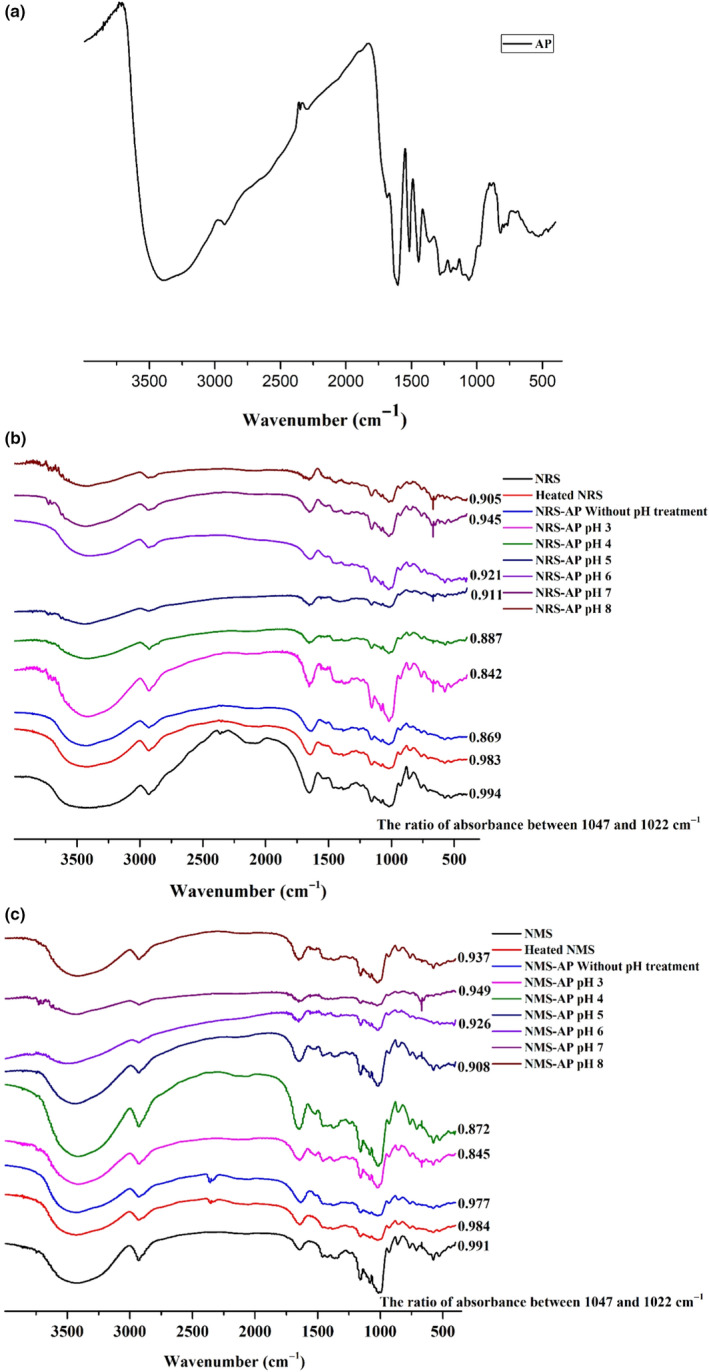
(a) FTIR spectra of AP; (b) FTIR spectra of NRS and NRS–AP mixtures under different pH conditions; (c) FTIR spectra of NMS and NMS–AP mixtures under different pH conditions

In the AP spectrum (Figure 2a), 3,400 cm^−1^ is the absorption peak of ‐OH, 2,920 cm^−1^ corresponds to the stretching vibration of ‐CH_2_, 1,444 cm^−1^ indicates the bending vibration of ‐CH, 1515 cm^−1^ is the stretching vibration of C‐C, 1,199 cm^−1^ and 1,060 cm^−1^ correspond to the stretching vibration of C‐O, and 1604 cm^‐1^ corresponds to the stretching vibration of carbonyl groups. In the NRS and NMS spectra (Figure 2b,c), 3,400 cm^‐1^ corresponds to the absorption peak of ‐OH, 2,929 cm^‐1^ corresponds to the stretching vibration of ‐CH_2_, 1,650 cm^‐1^ represents stretching vibration of ‐OH, which is related to water molecules in the amorphous region of starches, 1,047 cm^‐1^ corresponds to crystalline regions of starch, and 1,022 cm^‐1^ corresponds to amorphous regions of the starch (Chung, Liu, & Hoover, 2009).

No new absorption peaks were evident in the FTIR spectra for the starch–AP mixtures, indicating that no new chemical bonds formed as a result of these interactions even though the positions of certain peaks were changed to some extent. Certain peaks that were present in the polyphenol FTIR spectra were not present in the spectra for the starch–AP mixtures, which was attribute to the interaction between polyphenol and starch molecules (Liu et al., 2017). The ratio of absorbance between 1,047 cm^−1^ and 1,022 cm^−1^ was used to describe the degree of order (Liu et al., 2017). Starch–AP mixture ratio was lower than raw and heated starch, suggesting that AP could inhibit the retrogradation of NRS and NMS. Ratios of all the starch–AP mixture under various pH conditions were lower for raw and heated starch, and these starch–AP mixture ratios at low pH were lower than those at a high pH, suggesting that low pH could further inhibit the retrogradation of starch–AP mixtures, which is consistent with the results of our XRD analyses.

### 3.5 SEM analyses

**FIGURE 3:**
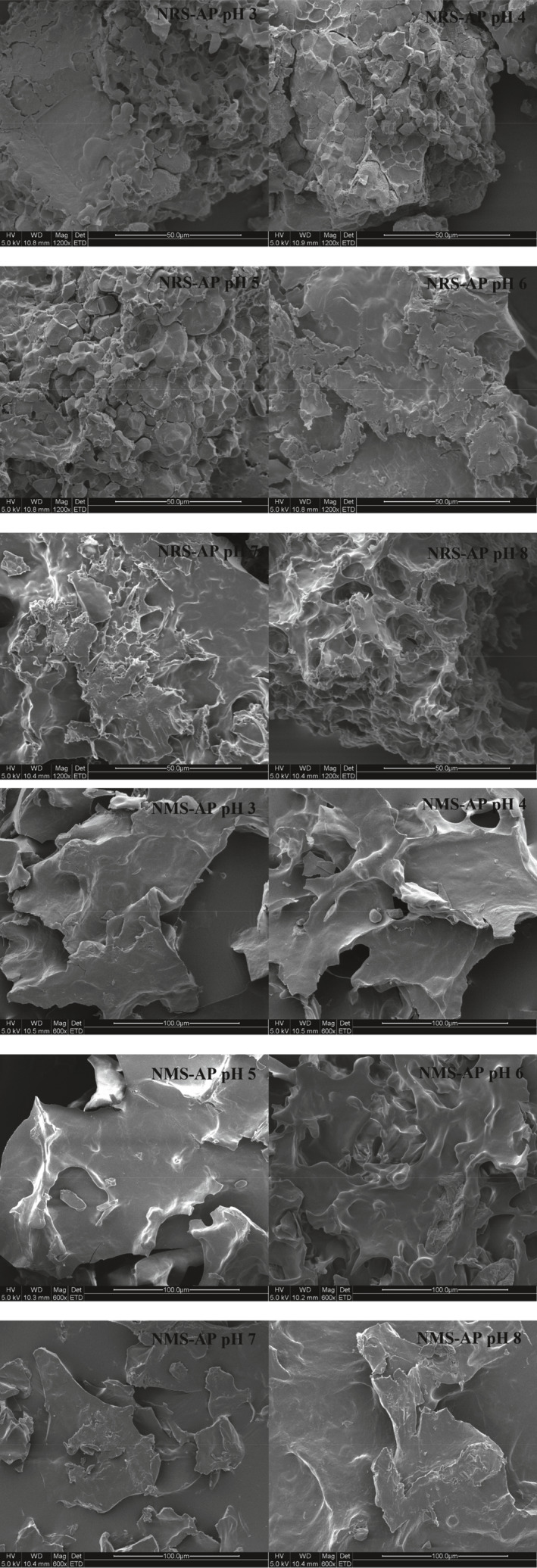
SEM assessment of NRS–AP and NMS–AP mixtures under a range of pH conditions

We next conducted *S*EM as a means of more directly assessing the properties of these starch–AP mixtures prepared under a range of different pH conditions (Figure 3). We found that pH had a significant impact on the structures of these NRS–AP and NMS–AP mixtures. Fragmentation was evident in NRS–AP structures treated under low pH conditions, consistent with the acid‐mediated hydrolytic cleavage of amylose molecules. Starch molecules combined with AP molecules to form a net‐like structure, and acid treatment did not appear to directly affect the interactions between NRS and AP. At a higher pH, large particles with a smooth surface and a mesh‐like structure were evident in NRS–AP mixtures, suggesting that alkaline conditions promoted NRS–AP complex agglomeration. Under acidic conditions, the NMS–AP mixture also exhibited distinct fragmentation similar to that observed in NRS–AP mixtures under low pH conditions, suggesting that acid‐mediated starch hydrolysis facilitates the generation of small molecules that can directly form complexes with polyphenols (Hedayati, Shahidi, Koocheki, Farahnaky, & Majzoobi, 2016). Under high pH conditions, NMS–AP particles appear to have a smooth surface, and no obvious mesh structure was observed. These findings together thus indicate that acidic conditions can reduce NRS–AP and NMS–AP particle size owing to reductions in starch molecular weight, whereas alkaline treatments can release the internal structure of starch and thereby allowing it to better interact with polyphenols (Hosseini‐Parvar, Osano, & Matia‐Merino, 2016).

Textural and functional properties are both important in starchy food, and as such we hypothesize that properties of starch–AP mixtures can be altered due to changes in pH conditions. Our future studies will, therefore, explore the effect of pH on the texture and functional properties of starch–AP mixtures while also assessing the structural consequences of these pH changes.

## 4 CONCLUSION

In this study, the impact of pH on the structural and physicochemical properties of NRS–AP and NMS–AP mixtures was investigated. The particle sizes of NRS–AP and NMS–AP mixtures decreased at low pH condition. A DSC analysis indicated that AP addition and low pH conditions could significantly inhibit retrogradation. XRD and FTIR analyses of starch–AP mixture intermolecular interactions, and crystal properties under different pH conditions revealed that AP interacted with starch chains to repolymerize the starch molecules, and low pH conditions could restrain the recrystallization and retrogradation of NRS and NMS. *SEM* analyses further revealed that fragmentation was evident under low pH conditions consistent with acid‐mediated removal of amylose molecules as was observed in our particle size distribution experiments. These results thus indicated that low pH could inhibit the retrogradation and reduce the particle sizes of NRS/NMS–AP mixtures, thereby improving the quality of starch–AP‐based foods.

## CONFLICT OF INTEREST

There are no conflicts of interest to declare.

## ETHICAL STATEMENT

There are no human or animal testing.
